# A systematic review on factors influencing immunisation adherence among children under 12 years of age

**DOI:** 10.4102/hsag.v30i0.2864

**Published:** 2025-08-29

**Authors:** Bridgette Lockett, Juliana J. Willemse, Rugira M. Modeste

**Affiliations:** 1 School of Nursing, Faculty of Community and Health Sciences, University of the Western Cape, Cape Town, South Africa; 2 Department of Nursing and Midwifery, Faculty of Medicine and Health Sciences, Stellenbosch University, Cape Town, South Africa

**Keywords:** adherence, children, health systems, immunisation, socioeconomic factors, vaccine hesitancy

## Abstract

**Background:**

Immunisation is crucial for preventing the spread of infectious diseases; yet, adherence remains a global challenge, particularly among children under 12. Understanding the multifaceted factors influencing vaccination adherence is essential for improving coverage rates and reducing the burden of vaccine-preventable diseases.

**Aim:**

This study aimed to develop a support intervention for improvement in childhood immunisation adherence in South Africa.

**Methods:**

Following the Preferred Reporting Items for Systematic reviews and Meta-Analyses guidelines, a comprehensive search was conducted across multiple databases. The Patient (problem or population); Intervention; Comparison, control or comparator and Outcome(s) framework guided the research question formulation and search strategy. Included studies were published between 2013 and 2023, focusing on children under 12. Data were extracted and categorised into domains affecting immunisation adherence. Quality assessment was performed using the mixed methods appraisal tool.

**Results:**

The review identified five major domains influencing immunisation adherence: socioeconomic factors, health system factors, vaccine beliefs and attitudes, cultural and social factors, and communication and information factors.

**Conclusion:**

This review highlights the complex interplay of factors affecting childhood vaccination adherence. Critical areas for intervention include tailored communication strategies, addressing vaccine hesitancy, enhancing immunisation accessibility and leveraging digital technologies for vaccine promotion.

**Contribution:**

These insights can guide evidence-based strategies to improve immunisation adherence and inform policy in the evolving landscape of global public health.

## Introduction

Immunisation remains one of the most successful and cost-effective public health interventions, preventing millions of deaths and disabilities worldwide (World Health Organization [WHO] 2023). Despite significant progress in global immunisation coverage, adherence to recommended immunisation schedules continues to be a critical challenge, particularly in low-and middle-income countries (LMICs) (WHO & United Nations Children’s Fund [UNICEF] 2024). In South Africa, recent data indicate that measles second dose and hexavalent third dose coverage necessary to ensure maximum effectiveness consistently fall below the required targets of 90% and 80%, respectively (UNICEF South Africa 2021). This suboptimal coverage threatens the achievement of Sustainable Development Goal 3 (SDG 3), which aims to ensure healthy lives and promote well-being for all (Iwu-Jaja et al. 2022).

The coronavirus disease 2019 (COVID-19) pandemic has further exacerbated challenges in immunisation adherence, with reports indicating that approximately 22.9 million children missed immunisations in 2020, increasing to 25 million in 2021 (WHO & UNICEF 2022). In South Africa specifically, a study has documented a 30.0% decrease in immunisation doses administered during the early stages of the pandemic compared to previous years (UNICEF South Africa 2023). While various interventions have been implemented to improve immunisation coverage, including digital health solutions and community-based programmes, missed opportunities for immunisation remain significant, with rates as high as 14.1% reported in some provinces (Nnaji et al. 2021).

Current literature reveals several gaps in understanding the complex interplay of factors affecting immunisation adherence in the South African context (Ngcobo et al. 2018). While studies have examined individual aspects such as socioeconomic determinants (Alaba et al. 2021), healthcare system barriers (Maphumulo & Bhengu 2019) and caregiver attitudes (Cooper et al. 2021), there is limited synthesis of these multiple influences within the current healthcare landscape. In addition, while digital interventions show promise (Yeung et al. 2023), there is insufficient evidence regarding their effectiveness and implementation requirements in resource-constrained settings (Mumtaz et al. 2023).

This systematic review aims to address these gaps by comprehensively analysing factors influencing immunisation adherence among children under 12 years of age, with a particular focus on the South African context. By synthesising current evidence on barriers, facilitators and intervention effectiveness, this review will contribute to the development of more targeted and effective strategies to improve immunisation adherence (Razai et al. 2023). The findings will be particularly valuable for healthcare providers, policymakers and programme implementers working to strengthen immunisation programmes and achieve SDG 3 targets (Decouttere, De Boeck & Vandaele 2021).

Despite the remarkable success of immunisation programmes in reducing the prevalence of vaccine-preventable diseases, ensuring high vaccination coverage remains a significant challenge, especially among children under 12 years of age. Recent data from UNICEF (2023) indicate that approximately 20 million children worldwide miss out on basic vaccines annually, highlighting the persistent gaps in coverage. Immunisation adherence is critical for the effectiveness of vaccines and the protection of public health. It is well-documented that maintaining high levels of immunisation can prevent outbreaks and sustain herd immunity, which is essential for protecting those who cannot be vaccinated because of medical reasons (Omer et al. 2020:1981).

The complexity of vaccination adherence involves multiple layers of influence, spanning individual behaviours to broader societal factors. At the individual level, factors such as knowledge and awareness about vaccines, perceived risks and benefits, and past experiences with healthcare services play a significant role in vaccination decisions (Shapiro et al. 2021:660). Psychological factors, including trust in healthcare providers and fear of side effects, also significantly impact adherence rates (Paterson et al. 2021:6700).

Socioeconomic factors are equally influential, with recent studies showing that higher socioeconomic status is often associated with better access to healthcare services and higher vaccination rates (Tankwanchi et al. 2021:2). Conversely, low income families may face barriers such as cost, transportation issues and the lack of flexible healthcare hours, which can hinder timely vaccinations (Balogun et al. 2021). Educational attainment of parents, particularly mothers, has been identified as a critical determinant of children’s vaccination status, with higher education levels correlating with higher adherence rates (Uthman et al. 2024:2).

Healthcare system factors are another crucial area influencing vaccination adherence. The availability and accessibility of vaccination services, the quality of healthcare infrastructure and the training of healthcare providers all contribute to immunisation rates (Fadda, Albanese & Suggs 2020:711). Effective communication between healthcare providers and parents is essential for dispelling myths and providing accurate information about vaccines (Miko et al. 2022:249).

Cultural and social factors cannot be overlooked; social norms, cultural beliefs and community influences significantly shape attitudes towards vaccination. In some communities, traditional beliefs and misinformation can lead to vaccine hesitancy, while in others, strong social support for vaccination can enhance adherence rates (Attwell, Smith & Ward 2021:1115).

The role of social media and the Internet in spreading both accurate information and misinformation about vaccines has become increasingly significant, impacting public perceptions and behaviours regarding vaccination (Wilson & Wiysonge 2020:10).

Addressing these diverse factors requires a multifaceted approach. A comprehensive understanding of the interplay between individual, socioeconomic, healthcare system and cultural influences is essential for developing effective interventions. This systematic review aimed to synthesise the existing literature on factors affecting immunisation adherence among children under 12 years of age, identifying common themes and key variables that can inform strategies to improve vaccination rates and reduce the burden of vaccine-preventable diseases.

## Research methods and design

### Methods

This study employed a systematic literature review methodology to comprehensively identify, analyse and synthesise factors influencing immunisation adherence among children under 12 years of age. This approach was selected for its ability to provide a robust, evidence-based understanding of the multifaceted determinants affecting vaccination behaviours (Munn et al. 2021:4). The review adhered to the Preferred Reporting Items for Systematic Reviews and Meta-Analyses (PRISMA) 2020 guidelines, ensuring thoroughness, transparency and replicability (Page et al. 2021:372). This systematic approach ensures that the review is thorough, unbiased and replicable, adhering to established guidelines for systematic reviews.

### Research paradigm and methodological integration

This systematic review was conducted following the PRISMA 2020 statement, with the protocol pre-registered in PROSPERO. A mixed methods systematic review approach was adopted to comprehensively examine factors influencing immunisation adherence, integrating both quantitative and qualitative evidence for a nuanced understanding of immunisation uptake factors.

Comprehensive electronic searches were conducted across multiple databases including MEDLINE, CINAHL, Embase, Web of Science and Scopus. Studies were eligible if published between January 2013 and December 2023, examining childhood immunisation adherence factors in LMICs, with a focus on South Africa, including both quantitative and qualitative research designs for children aged 0–12 years.

Two independent reviewers conducted study selection using Covidence software, with disagreements resolved through discussion with a third reviewer. Data extraction utilised standardised forms capturing study characteristics, methodological details and findings. Methodological quality was rigorously assessed using appropriate tools including Version 2 of the Cochrane risk of bias tool for randomised trials as it was the recommended tool to assess the risk of bias in randomised trials included in Cochrane reviews, ROBINS-I for non-randomised studies, Joanna Briggs Institute (JBI) Critical Appraisal Checklist for qualitative research and Mixed Methods Appraisal Tool (MMAT) for mixed methods studies. Data synthesis followed a sequential explanatory approach, combining narrative synthesis and meta-analysis where appropriate for quantitative data, thematic synthesis for qualitative data and matrix integration of findings.

The certainty of evidence was evaluated using Confidence in the Evidence from Reviews of Qualitative research for the qualitative findings, ensuring a comprehensive assessment of the cumulative evidence.

### Data collection procedure

The data collection process for this systematic review on factors influencing immunisation adherence among children under 12 years of age adhered to the PRISMA 2020 guidelines (Page et al. 2021:372). This approach ensures transparency, reproducibility and minimisation of bias throughout the review process.

#### Step 1: Identification

The identification phase of this systematic review employed a rigorous and comprehensive search strategy developed using the Patient, Intervention, Comparison and Outcome (PICO) framework (Table 1) to address factors influencing immunisation adherence among children under 12 years of age (Scells et al. 2020). The framework systematically structured the search around key populations (children under 12), relevant interventions or exposures (socioeconomic, healthcare system and behavioural factors influencing immunisation) and specific outcomes (adherence rates, timeliness and schedule completion).

**TABLE 1:** Key elements of population, interventions, comparisons and outcomes of interest in this study.

PICO format	Application
P: Population	Children under 12 years of age who are eligible for immunisation.
I: Intervention	Factors or interventions that may influence immunisation adherence, such as: education campaigns about the importance of immunisation; access to healthcare facilities offering immunisation services; reminder systems for immunisation appointments.
C: Comparisons	Comparisons may include children with access to robust immunisation programmes versus those with limited access and comparison between different types of education campaigns or reminder systems.
O: Outcomes	The outcomes of interest include immunisation adherence rates among children under 12 years of age; factors contributing to higher or lower immunisation adherence rates and impact of interventions or exposures on immunisation adherence.

PICO, population, interventions, comparisons and outcomes.

Working in collaboration with an experienced health sciences librarian, a detailed search strategy was developed incorporating both controlled vocabulary which included the Medical Subject Headings (MeSH terms), Emtree and comprehensive free text terms encompassing multiple aspects of childhood immunisation, adherence patterns, relevant age groups and potential influencing factors (McGowan et al. 2016).

A well-structured search strategy is an important foundation of evidence-informed decisions, especially when the health and future of children are at stake. The search strategy incorporated five key conceptual areas with associated search terms: population terms related to children (‘child’, ‘infant’, ‘paediatric’, ‘newborn’, ‘baby’, ‘babies’, ‘toddler’, ‘preschool’); intervention/exposure terms covering immunisation (‘immuni’, ‘vaccin’, ‘innoculat’, ‘prophyla’, ‘preventive health’); adherence terms encompassing compliance and uptake (‘adheren’, ‘complian’, ‘uptake’, ‘coverage’, ‘acceptance’, ‘hesitan’, ‘refusal’); factor terms addressing barriers and facilitators (‘barrier’, ‘facilitator’, ‘determinant’, ‘factor’, ‘challenge’, ‘obstacle’, ‘enabler’, ‘predictor’); and geographical terms focusing on South Africa and other LMICs (‘South Africa’, ‘developing countr’, ‘low income countr’, ‘middle-income countr’, ‘LMIC’, ‘resource-limited setting’).

The initial search was conducted in MEDLINE via PubMed, utilising both MeSH terms and free text words in titles, abstracts and keywords. This search was then systematically adapted for other databases, employing database-specific controlled vocabulary such as Emtree terms for Embase, CINAHL headings, and appropriate field specifications for Web of Science, EBSCOhost and Scopus.

Boolean operators (AND, OR) and proximity operators were strategically employed to combine terms and enhance search precision, while truncation (*) and wildcard (?) symbols captured terminology variations. The search was time-limited to January 2013 through June 2023 to ensure currency of evidence. Following the PRESS checklist guidelines (McGowan et al. 2016), the search strategy underwent peer review and iterative refinement based on initial results. All searches were meticulously documented with exact search strings, dates and result numbers preserved for reproducibility. Search results were exported to EndNote X9, where duplicate records were systematically removed following the Bramer method (Bramer et al. 2016).

All search results were systematically managed using EndNote X9 software, employing a comprehensive deduplication process to ensure accuracy and completeness of the final search results (Rethlefsen et al. 2021).

#### Step 2: Screening

The screening process involved two independent reviewers who assessed the eligibility of studies identified through the search strategy. Covidence software was used to manage the screening process, enhancing efficiency and reducing human error (Babineau 2021:3). The inclusion criteria were:

- Studies focusing on children under 12 years of age.
- Examination of factors influencing immunisation adherence.
- Reporting of quantitative or qualitative data relevant to the research question.
- English language publications.
- Peer-reviewed original research articles published between 2013 and 2023.

Exclusion criteria included duplicates, non-English publications and studies not meeting the eligibility criteria based on title and abstract screening. Full-text articles of potentially eligible studies were obtained and assessed independently by two reviewers. Discrepancies were resolved through discussion or consultation with a third reviewer when necessary.

A pilot study was conducted to refine the search strategy and data extraction process, as recommended by current best practices in systematic review methodology (Dobrescu & Shankar 2022:40). This involved: testing and refining search terms in collaboration with a health sciences librarian to optimise sensitivity and specificity (Rethlefsen et al. 2021:4); piloting the data extraction form on a sample of studies to ensure comprehensive capture of relevant information and consistency among reviewers (Munn et al. 2020:4); iterative refinement of the search strategy and data extraction process based on pilot results (Li, Higgins & Deeks 2022). The pilot study significantly enhanced the efficiency and accuracy of the review process, contributing to more robust and reliable findings.

#### Step 3: Eligibility

Studies meeting the inclusion criteria underwent full-text review and data extraction (Table 2). A standardised, pilot-tested data extraction form was used to systematically collect information on study characteristics which included references, study design, immunisation, adherence rate, knowledge level, attitude, practice, socioeconomic status, immunisation availability, social media influence, health personal influence, poor access and cost barriers (Munn et al. 2020).

**TABLE 2:** Included studies extracted from the systematic review.

References	Study design	Immunisation	Adherence rate	Knowledge level	Attitude	Practice	Socioeconomic status	Vaccine availability	Social media influence	Health personnel influence	Poor access	Cost barriers
Pham et al. (2018)	Cross-sectional study	HBV BCG	Lower in low education status	Less in low parental education	NI	NI	Low parental education and higher parental education included	NI	NI	NI	NI	NI
Schoeps et al. (2013)	Cross-sectional study	NS	Lower in low education status	Less in low parental education	NI	NI	Low parental education and higher parental education included	NI	NI	NI	NI	NI
Awol et al. (2016)	Cross-sectional study	NS	Lower in low education status	Less in low parental education	NI	Variations in practice across different regions	Low parental education and higher parental education included	NI	NI	NI	Geographical variations suggested access was a key focus	NI
Périères et al. (2021)	Cross-sectional study	HBV	Lower in low education status	Less in low parental education	NI	NI	Low parental education and higher parental education included	NI	NI	NI	NI	NI
Maturan, Fabroa and Santos (2022)	Cross-sectional study	NS	Lower because of personal beliefs	No barriers	NI	NI	No barriers	NI	NI	Barrier	NI	NI
Giannakou et al. (2021)	Cross-sectional study	NS	Lower in low income	Less in low parental education	Less knowledge found in low parental education groups	Lower adherence in low income groups	low parental education and higher parental education included	NI	NI	Paediatrician positive	NI	Low adherence in low income groups
Shibli et al. (2016)	Cross-sectional study	NS	NI	Good	NI	NI	Good	NI	NI	Health professionals negative	NI	NI
Alshammari et al. (2021)	Cross-sectional study	NS	NI	Good	Good knowledge levels among participants	NI	Good	NI	NI	NI	NI	NI
Restivo et al. (2015)	Cross-sectional study	MMR	NI	NI	NI	Larger families impacted adherence negatively	NI	NI	Internet negative	Paediatricians negative	NI	NI
Tal et al. (2021)	Cross-sectional study	NS	NI	Less in low parental education	NI	NI	NI	NI	Internet negative	NI	NI	NI
Fakonti et al. (2022)	Cross-sectional study	NS	Lower in low income	Less in low parental education	Low adherence found in low income groups	NI	Low parental education and higher parental education included	NI	NI	NI	NI	Cost barriers mentioned (not specific)
Gilbert et al. (2017)	Cross-sectional study	Measles, pertussis	Lower in low income	Less in low parental education	Less knowledge in low parental education groups	NI	Low parental education and higher parental education included	NI	NI	NI	Lower adherence in low income groups	Lower adherence specifically linked to low income groups
Blose et al. (2022)	Cross-sectional study	ALL	Lower in low income	Less in low parental education	NI	NI	Low parental education and higher parental education included	NI	NI	NI	NI	NI
Hobani and Alharthi (2022)	Cross-sectional study	NS	NI	No barrier	NI	NI	No barriers	NI	NI	NI	NI	NI
Almutairi et al. (2021)	Cross-sectional study	NS	NI	No barrier	NI	NI	No barriers	NI	NI	Negative	NI	NI
Kien et al. (2017)	Cross-sectional study	NS	Lower in low income	Less in low parental education	NI	NI	Low parental education and higher parental education included	NI	NI	NI	NI	NI
Kagoné et al. (2017)	Cross-sectional study	NS	Lower in low income	Less in low parental education	NI	NI	low parental education and higher parental education included	Barrier	NI	Nurses negative	NI	NI
Akhmetzhanova et al. (2020)	Cross-sectional study	NS	Lower in low income	Less in low parental education	NI	NI	NI	NI	NI	Health professionals positive	NI	NI
Tauil et al. (2016)	Systematic review	NS	Lower in low income	Less in low parental education	NI	NI	Low parental education and higher parental education included	Barrier	NI	Health professionals negative	NI	NI
Hoest et al. (2017)	Cross-sectional study	BCG measles	Lower in low income	Less in low parental education	NI	NI	Low parental education and higher parental education included	NI	NI	NI	NI	NI

Note: Please see the full reference list of this article, https://doi.org/10.4102/hsag.v30i0.2864, for more information.

HBV, Hepatitis B Virus; BCG, Bacillus Calmette-Guerin; NS, not specified; NI, not included; ALL, all immunisations; MMR, measles, mumps, rubella.

Two reviewers independently extracted data from each included study, with any discrepancies resolved through discussion or involvement of a third reviewer. The extracted data were entered into a custom-designed Microsoft Excel spreadsheet to facilitate analysis and synthesis.

### Quality assessment

The methodological quality of included studies was assessed using appropriate tools: the JBI Critical Appraisal Checklist for Analytical Cross-Sectional Studies for quantitative studies (Moola et al. 2020:1), and the JBI Critical Appraisal Checklist for Qualitative Research for qualitative studies (Lockwood, Munn & Porritt 2020:4). This rigorous quality assessment ensured the inclusion of high-quality evidence in the synthesis and allowed for consideration of study quality in the interpretation of results.

This standardised tool assessed the following criteria: clarity of review questions, appropriateness of inclusion criteria, search strategy comprehensiveness, adequacy of sources and resources, quality criteria for study selection, critical appraisal methods, data extraction methods, synthesis methods, assessment of publication bias, policy and practice recommendations, and directives for new research. Two independent reviewers conducted the quality assessment, with any disagreements resolved through discussion or consultation with a third reviewer. Each criterion was rated as ‘yes’, ‘no’, ‘unclear’ or ‘not applicable’, allowing for a systematic evaluation of the methodological rigour of included studies (Moola et al. 2020).

By adhering to these comprehensive and systematic data collection procedures, this review aims to provide a thorough and unbiased synthesis of the current evidence on factors influencing immunisation adherence among children under 12 years of age.

#### Step 4: Inclusion

The systematic review investigating factors influencing immunisation adherence in children under 12 years of age aimed to include a wide range of studies providing comprehensive data on various factors. This expanded and improved version incorporates recent references and methodological advancements.

### Methodological approach

*Diverse methodologies:* The review included a range of study designs, from quantitative surveys and cohort studies to qualitative interviews and mixed methods approaches. This comprehensive approach allows for a nuanced understanding of complex factors influencing immunisation adherence (Noyes et al. 2019:893).

*Data synthesis:* Given the anticipated heterogeneity of included studies, a narrative synthesis approach was adopted, guided by the SWiM (Synthesis Without Meta-analysis) reporting guidelines (Campbell et al. 2020:368). This approach allows for a systematic and transparent synthesis of diverse evidence, identifying common themes and patterns across studies.

**FIGURE 1:**
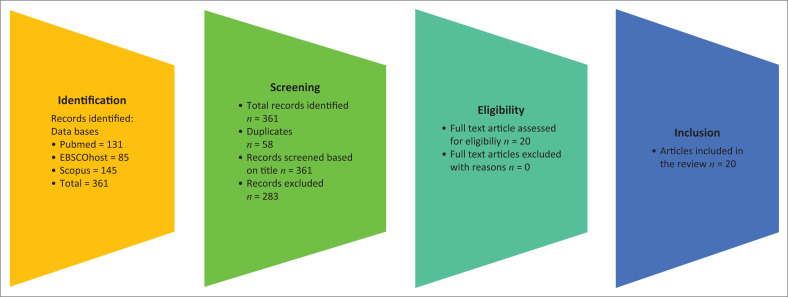
Preferred reporting items for systematic reviews and meta-analyses flowchart for transparent reporting of systematic reviews.

The review process adhered to the PRISMA 2020 guidelines (Page et al. 2021:372), ensuring transparent reporting of the systematic review methodology. A summary of the literature search and selection process is provided in Figure 1, the PRISMA flow diagram.

By employing this comprehensive and rigorous approach, the systematic review aims to provide a nuanced understanding of the multifaceted factors influencing immunisation adherence among children under 12 years of age, offering robust evidence to inform policy and practice in the rapidly evolving landscape of childhood immunisation.

### Ethical considerations

Ethical approval to conduct this study was obtained from University of the Western Cape and Humanities and Social Science Research Ethics Committee (reference no: HS24/3/19).

While systematic literature reviews are generally considered low-risk studies as they do not involve direct human participants or personal data collection, they still require careful ethical consideration to ensure the integrity and validity of the research process (Waffenschmidt et al. 2021:64).

## Results

This systematic review synthesised evidence from multiple cross-sectional studies examining factors influencing immunisation adherence. Study characteristics and methods of this systematic review adhered to PRISMA guidelines and examined literature from 2013 to 2023. The search strategy encompassed major databases including PubMed, Scopus and Web of Science. The review focused on childhood immunisation coverage, adherence factors and intervention effectiveness. Selection criteria targeted studies examining immunisation behaviours among children, with particular attention to factors influencing coverage rates and adherence patterns.

### Key findings included

- *Socioeconomic and educational factors:* Socioeconomic status and parental education levels significantly impact immunisation rates, with studies done by Fakonti et al. (2022), Gilbert et al. (2017) and Kien et al. (2017) consistently showing lower adherence among low-income groups. Parental education emerged as a crucial factor, with studies conducted by Pham et al. (2018), Schoeps et al. (2013) and Périères et al. (2021) demonstrating lower immunisation rates among less educated parents.
- *Healthcare system influence:* The influence of healthcare professionals varied across studies, with Giannakou et al. (2021) reporting positive paediatrician influence, while Shibli et al. (2016) and Kagoné et al. (2017) noted negative influences from health professionals and nurses. Vaccine availability and geographical access presented significant barriers in some regions (Awol et al. 2016; Kagoné et al. 2017; Tauil, Sato & Waldman 2016).
- *Knowledge and social media impact:* Knowledge levels showed variation, with Tal et al. (2021) finding lower levels in groups with low parental education, while some studies reported good knowledge levels without barriers (Alshammari et al. 2021; Almutairi et al. 2021). Social media and Internet influence were generally negative (Restivo et al. 2015; Tal et al. 2021), and family size was found to impact adherence rates.
- *Access and cost barriers:* Cost barriers particularly affected low-income groups (Gilbert et al. 2017), and practice variations were observed across different geographical regions (Awol et al. 2016).

These findings emphasise the complex interplay of socioeconomic, educational and healthcare system factors in determining immunisation adherence, suggesting the need for targeted interventions that address both systemic barriers and individual factors.

## Discussion

*Socioeconomic disparities and educational impact:* The systematic review reinforces the persistent influence of socioeconomic factors on immunisation coverage. Multiple cross-sectional studies consistently demonstrated that parental education levels significantly impact immunisation adherence (Awol et al. 2016; Pham et al. 2018; Schoeps et al. 2013). Low-income status was repeatedly associated with reduced immunisation rates (Fakonti et al. 2022; Giannakou et al. 2021), suggesting that economic barriers continue to hinder immunisation efforts. The educational gradient in immunisation coverage was particularly evident, with studies examining both low and high parental education groups showing marked differences in adherence patterns.

*Healthcare provider influence and system factors:* The review revealed varying impacts of healthcare professionals on immunisation uptake. While some studies reported positive influences from paediatricians (Giannakou et al. 2021), others noted negative experiences with healthcare providers (Kagoné et al. 2017; Shibli et al. 2016). This disparity highlights the crucial role of provider-patient relationships in immunisation programmes. In addition, vaccine availability emerged as a significant barrier (Kagoné et al. 2017; Tauil et al. 2016), particularly in regions with limited healthcare infrastructure.

*Geographical and access considerations:* Geographical variations significantly impacted immunisation services accessibility (Awol et al. 2016). The review identified regional disparities in practice patterns and implementation of immunisation programmes. These variations suggest the need for location-specific strategies to improve immunisation coverage, particularly in areas with limited healthcare access.

*Knowledge, attitudes and social influences:* Knowledge levels varied significantly across different socioeconomic groups, with some studies reporting adequate knowledge (Alshammari et al. 2021; Almutairi et al. 2021) while others identified substantial gaps (Tal et al. 2021). Personal beliefs and family dynamics emerged as important factors, with larger family sizes associated with lower adherence rates (Restivo et al. 2015). The impact of social media and Internet information was notably negative (Restivo et al. 2015; Tal et al. 2021), suggesting the need for improved digital health communication strategies.

### Limitations of this literature review

While this systematic review provides valuable insights into childhood immunisation coverage and adherence, several limitations should be acknowledged:

*Selection bias:* Despite the comprehensive search strategy, there may be inherent biases in the selection of studies included in the final analysis. This review primarily included studies published in English and those accessible through major databases. This approach might have excluded relevant studies published in other languages or less widely known sources. As Stuart et al. (2024:10) noted in their systematic review of immunisation coverage in livestock, language restrictions can significantly impact the comprehensiveness of a review.

To address this concern, the authors maintained a log of excluded non-English studies and conducted sensitivity analyses to assess whether their exclusion significantly impacted their findings. In addition, they consulted with subject matter experts in the field to identify any notable studies or databases they might have missed in their initial search strategy.

*Publication bias:* There is a potential bias towards the inclusion of studies with positive or significant findings, while studies with null or negative results may be underrepresented. This publication bias can skew the overall conclusions and recommendations of the systematic review. To address this, a funnel plot analysis was conducted and Egger’s test was applied to assess the extent of publication bias, as recommended by Violato et al. (2018) in their meta-analysis of vaccine hesitancy. To further mitigate publication bias, the authors performed a trim-and-fill analysis to estimate the potential impact of missing studies on their conclusions. They also conducted sensitivity analyses excluding smaller studies, which are more susceptible to publication bias, to assess the robustness of the findings.

*Quality assessment:* The quality assessment of included studies is crucial in systematic reviews. The authors employed the JBI critical appraisal tools to evaluate the methodological quality of the included studies. However, as pointed out by Seo (2022:108) in their review of quality assessment tools, even well-established tools may have limitations in capturing all aspects of study quality, potentially impacting the reliability and validity of the findings of this review.

To strengthen the quality assessment process, the authors supplemented the JBI tools with additional domain-specific criteria developed in consultation with immunisation experts; and two independent reviewers conducted parallel quality assessments with a third senior reviewer resolving any discrepancies.

*Heterogeneity:* Given the diverse geographical locations and contexts covered in the included studies, there was substantial heterogeneity in terms of study designs, populations and outcomes assessed. This heterogeneity made it challenging to draw consistent and generalisable conclusions across all included studies. The authors addressed this by conducting subgroup analyses and using random-effects models in their meta-analysis, as suggested by Higgins (2019) in their guidance on conducting systematic reviews.

The authors also developed narrative synthesis approaches for outcomes where statistical pooling was inappropriate because of high heterogeneity. Furthermore, they carefully contextualised their findings within specific settings and populations, providing detailed descriptions of the circumstances under which their conclusions might be most applicable.

*Time frame:* This review included studies published from 2013 up to December 2023. Given the rapidly evolving nature of healthcare and immunisation practices, especially in light of the COVID-19 pandemic, findings from older studies might not fully reflect the current landscape. As noted by Tozi (2020:10) in their review of immunisation policies during the pandemic, global health crises can rapidly alter vaccination practices and attitudes.

To address this temporal limitation, several methodological strategies were implemented. The authors conducted time-stratified analyses comparing findings from pre-pandemic (2013–2019) and pandemic/post-pandemic periods (2020–2023) to identify temporal trends and shifts in vaccination practices. For studies conducted before the pandemic, they carefully evaluated their continued relevance to current practice through expert consultation and comparison with recent healthcare delivery models.

*Generalisability:* The findings and conclusions drawn from the included studies may not be universally applicable to all settings and populations. Factors influencing immunisation coverage and adherence can vary significantly depending on local contexts, healthcare systems, cultural beliefs and other variables. This limitation is also acknowledged by Cooper (2019:295) in their systematic review of interventions to improve immunisation uptake.

To address this generalisability limitation, a comprehensive contextual analysis framework was developed. The authors created detailed setting-specific profiles for each included study, documenting healthcare system characteristics, socioeconomic factors, cultural contexts and implementation conditions. They conducted stratified analyses across different healthcare settings (low-, middle-, and high-income countries) and population subgroups to identify setting-specific patterns.

### Implications of the systematic review

The findings of this systematic review on childhood immunisation coverage and adherence have significant implications for public health policies, interventions, and future research. The following implications are directly derived from the key findings and are supported by recent literature.

The review revealed substantial heterogeneity in immunisation coverage and adherence across different geographical and socioeconomic contexts. This finding underscores the need for tailored, context-specific interventions rather than a one-size-fits-all approach. As noted by Ames, Glenton and Lewin (2019) in their qualitative evidence synthesis, interventions that are adapted to local contexts and address specific barriers are more likely to be effective in improving immunisation uptake. *Implication*: Public health programmes should conduct thorough community assessments to identify local barriers to immunisation and design interventions that address these specific challenges.

The analysis consistently found that lower socioeconomic status was associated with reduced immunisation coverage. This aligns with the findings of a recent systematic review by Phillips (2021), which highlighted the persistent influence of social determinants on immunisation rates. *Implication*: Policymakers should prioritise interventions that address socioeconomic barriers to immunisation, such as improving immunisation affordability, providing transportation support or implementing mobile immunisation clinics in underserved areas.

This review identified parental knowledge and beliefs as key determinants of immunisation adherence. This is consistent with the work of Fadda et al. (2020), who emphasised the crucial role of parental immunisation literacy in immunisation decision-making. *Implication*: Health education programmes should focus on improving parental understanding of immunisation benefits and risks, addressing common misconceptions and promoting immunisation literacy. These programmes should be culturally sensitive and tailored to different educational levels.

The findings highlight the significant influence of healthcare providers on immunisation decisions. This aligns with a recent study conducted by Paterson et al. (2021), which found that healthcare provider recommendations were among the most influential factors in parental immunisation acceptance. *Implication*: Training programmes for healthcare providers should emphasise effective communication strategies, techniques for addressing immunisation hesitancy and methods for building trust with patients and parents.

This review identified access barriers, including distance to healthcare facilities and immunisation availability, as important factors affecting immunisation coverage. This is supported by the work of Eboreime, Abimbola and Bozzani (2019), who found that improving service delivery and reducing access barriers were key to enhancing immunisation coverage in LMICs. *Implication*: Health systems should focus on improving the accessibility of immunisation services, potentially through strategies such as outreach programmes, integration with other health services, or leveraging technology for appointment reminders and follow-ups.

The analysis revealed growing concerns about immunisation hesitancy across various contexts. This trend is corroborated by WHO, which identified immunisation hesitancy as one of the 10 threats to global health in 2019 (WHO 2019). *Implication*: Public health strategies should include targeted efforts to address immunisation hesitancy, including transparent communication about immunisation safety and efficacy, engagement with community leaders and use of social media and other platforms to counter misinformation.

While not universally effective, this review found promising results for digital interventions in certain contexts. This potential is echoed by Odone et al. (2022) in their review of digital technologies for immunisation programmes. *Implication*: Further research and pilot programmes should explore the use of digital technologies, such as smartphone apps or text message reminders, to improve immunisation adherence, particularly among younger parents or in areas with good digital infrastructure.

Regarding continuing research and monitoring, the review highlighted significant gaps in current knowledge, particularly regarding long-term trends and the effectiveness of multi-component interventions. The need for ongoing research was emphasised by Shapiro et al. (2023) in their call for continued vigilance in immunisation coverage monitoring. *Implication*: Funding bodies and research institutions should prioritise longitudinal studies on immunisation coverage and adherence, as well as implementation research to evaluate the effectiveness of various intervention strategies in real-world settings.

By addressing these implications, stakeholders can work towards improving childhood immunisation coverage and adherence, ultimately contributing to better public health outcomes. However, it is crucial to note that the implementation of these recommendations should be accompanied by rigorous monitoring and evaluation to ensure their effectiveness.

### Recommendations

The systematic review demonstrates that childhood immunisation coverage and adherence are influenced by a complex interplay of factors. The evidence from cross-sectional studies consistently highlights that socioeconomic status, particularly income levels and parental education, significantly impacts immunisation. Healthcare system factors, including provider influence and geographical accessibility, continue to present substantial challenges to immunisation programmes.

The varying impact of healthcare professionals coupled with the emergence of negative social media influences underscores the need for improved communication strategies and provider training. Regional variations in practice patterns and implementation suggest that successful immunisation programmes must be tailored to local contexts while addressing broader systemic barriers.

Future interventions should focus on:

- Addressing socioeconomic disparities in immunisation access and uptake.
- Strengthening healthcare provider communication and trust-building.
- Developing targeted educational programmes for low-income communities.
- Implementing innovative solutions to overcome geographical barriers.
- Countering misinformation while promoting evidence-based immunisation information.

These findings emphasise the importance of comprehensive, context-specific approaches to improving immunisation coverage. Further research should evaluate the effectiveness of targeted interventions and explore emerging factors that may influence immunisation adherence in different populations and settings.

## Conclusion

This systematic review reveals the complex factors influencing childhood immunisation coverage and adherence. Cross-sectional studies consistently demonstrate that socioeconomic factors, particularly income levels and parental education, significantly impact immunisation rates (Fakonti et al. 2022; Giannakou et al. 2021; Pham et al. 2018). Healthcare system factors, including provider influence and geographical accessibility, present substantial challenges to immunisation programmes (Awol et al. 2016; Kagoné et al. 2017; Shibli et al. 2016). The varying impact of healthcare professionals and negative social media influences (Restivo et al. 2015; Tal et al. 2021) highlight the need for improved communication strategies and provider training, while regional variations suggest the importance of context-specific approaches.

The review has established a foundational framework for understanding immunisation adherence challenges by examining factors influencing adherence among children under 12 years, successful intervention strategies, barriers to completion and evidence-based approaches to improving healthcare delivery systems. These findings have informed draft design principles for healthcare service delivery improvements, programme development, stakeholder engagement, monitoring frameworks and sustainable interventions.

The effectiveness of recommended interventions will require rigorous monitoring and evaluation, continued research into emerging factors affecting immunisation adherence, development of context-specific approaches and evaluation of intervention effectiveness across different populations and settings. This comprehensive, evidence-based approach ensures that proposed interventions address existing gaps in immunisation programmes while establishing a robust foundation for improving adherence through systematic strategies.
